# Supplementary material for: Infant microbiome cultivation and metagenomic analysis reveal Bifidobacterium 2’-fucosyllactose utilization can be facilitated by coexisting species
Source: Nat Commun. 2023 Nov 16;14:7417. doi: 10.1038/s41467-023-43279-y (PMC10654741; doi:10.1038/s41467-023-43279-y)
Supplement: Supplementary file 1 — Supplementary information [file 41467_2023_43279_MOESM1_ESM.pdf]

Supplementary Information

Infant microbiome cultivation and metagenomic analysis reveal *Bifidobacterium* 2’-fucosyllactose utilization can be facilitated by coexisting species

Lou et al.

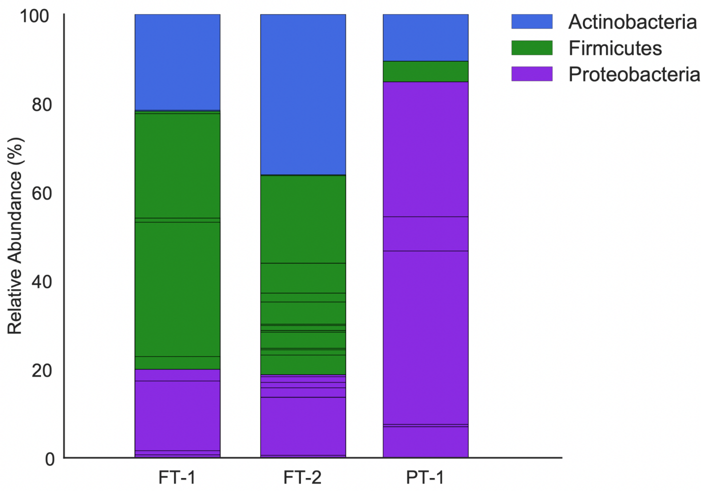

Supplementary Figure 1. Community compositions of the three infant stool inocula

The x-axis represents the stool inocula. Bar height represents normalized species relative abundance, and bars are colored by phylum. Sections of the same color with horizontal black lines correspond to individual species of the same phylum. Source data are provided as a Source Data file.

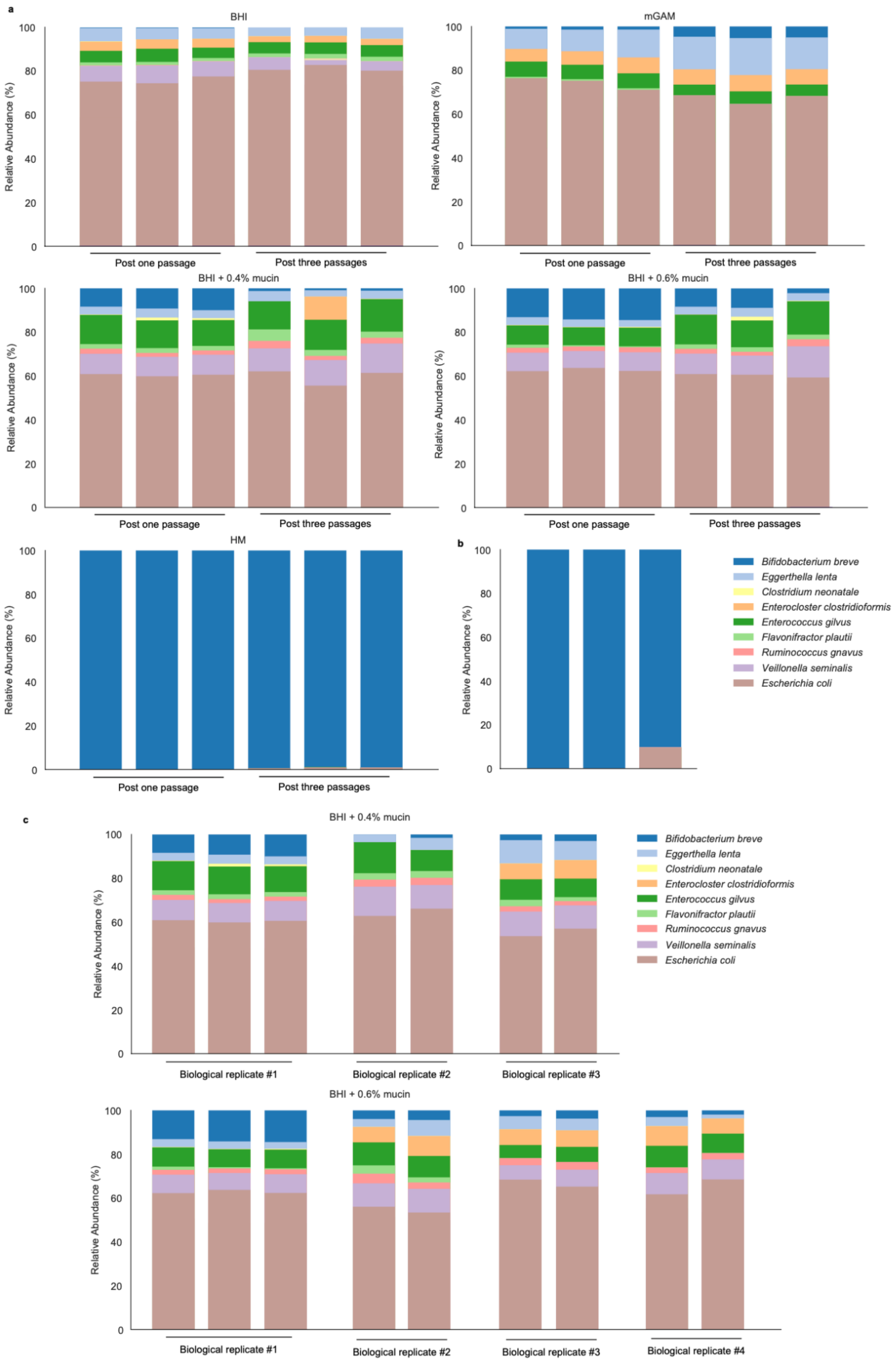

Supplementary Figure 2. Stability and Reproducibility of the FT-1 *in vitro* microbiomes

a FT-1 in different growth media, and the compositions are compared between the first and the third passaging (x-axis, n = 3 technical replicates for each independent experiment). Bar height represents normalized species relative abundance, and bars are colored by species. **b** An HM-enrichment glycerol inoculum was grown in BHI + 0.6% mucin (n=3 technical replicates for each independent experiment). **c** Reproducible FT-1 microbiome cultivation with BHI + 0.4% or 0.6% mucin. FT-1 inocula were inoculated in BHI + 0.4% or 0.6% mucin in three or four independent experiments (n = 2 or 3 technical replicates for each independent experiment) with one passage. Source data are provided as a Source Data file.

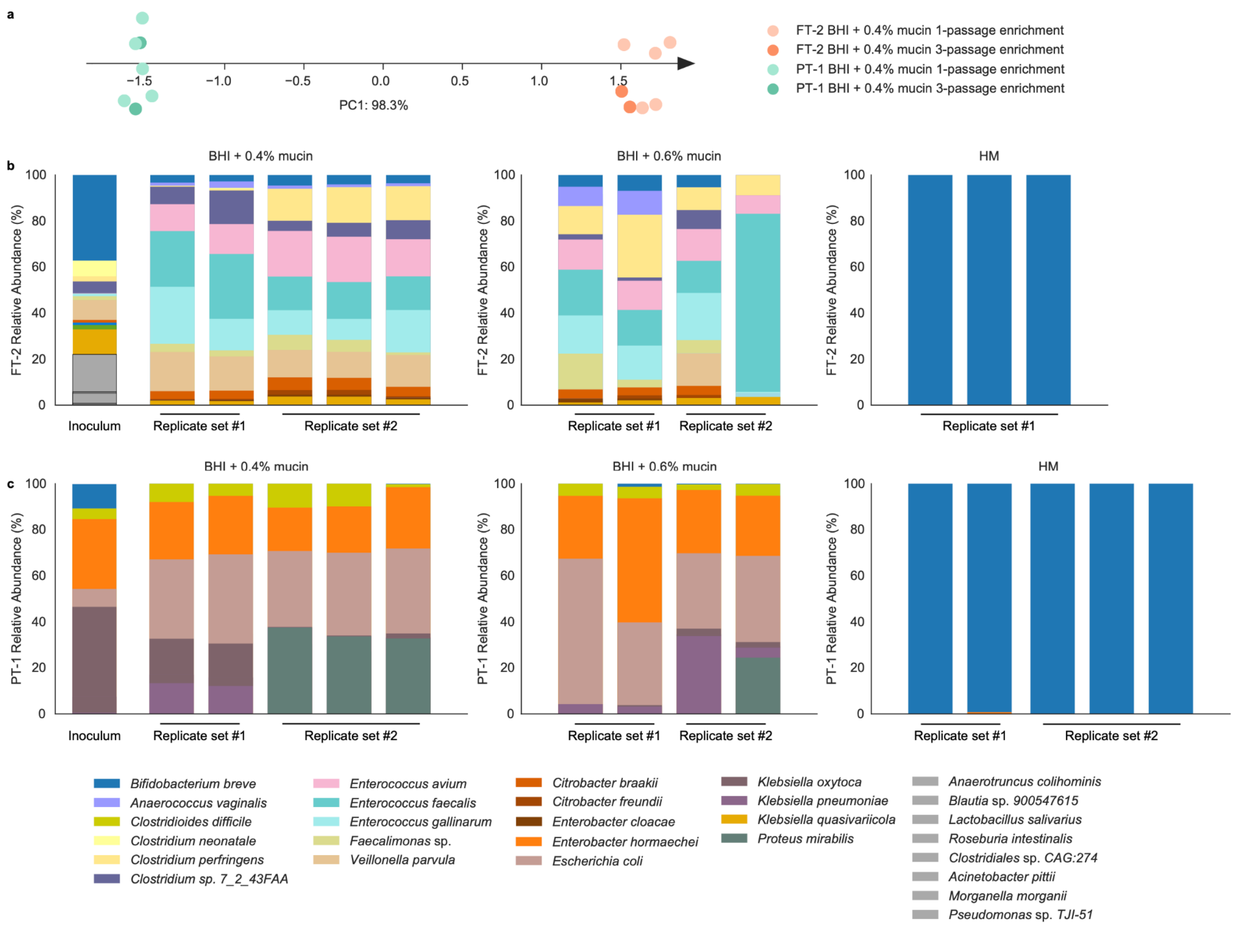

Supplementary Figure 3. Inoculation of two additional infant gut microbiomes

a PCA calculated using the weighted UniFrac Distances comparing FT-2 (green) and PT-1 (orange) communities grown in BHI + 0.4% mucin over three passages. Each circle represents a replicate (n = 2 or 3 technical replicates for each independent experiment). **b-c** FT-2 (**b**) and PT-1 (**c**) inocula were inoculated in BHI + 0.4%, BHI + 0.6% mucin, or HM, with one passage. Bar height represents normalized species relative abundance, and bars are colored by species. Organisms colored in gray (N=8 organisms) were present in the FT-2 stool inoculum but not in any cultivated communities. All organisms in PT-1 were detected in cultivated communities. The x-axis represents technical replicates (n = 2 or 3 technical replicates for each independent experiment). Source data are provided as a Source Data file.

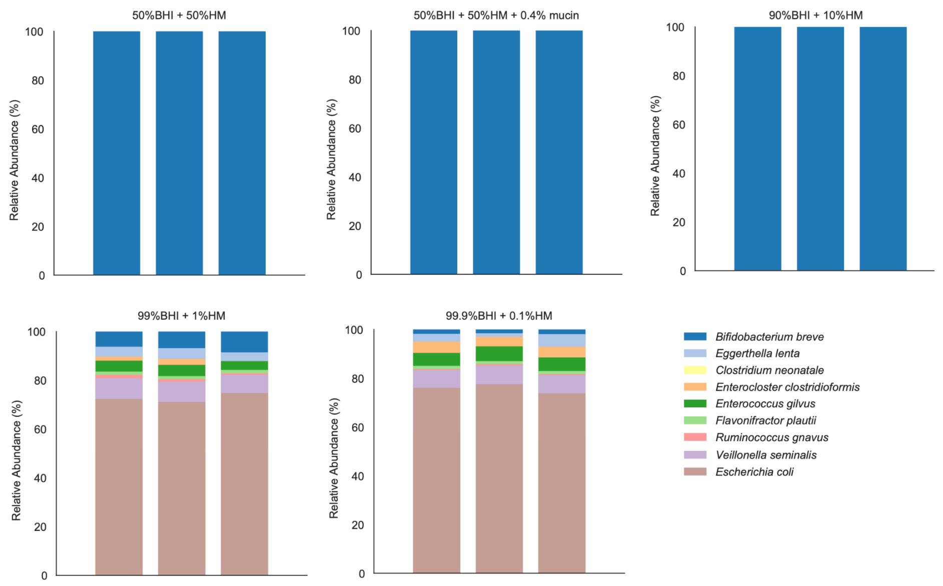

Supplementary Figure 4. FT-1 microbiome compositions in BHI-HM mixtures

FT-1 inocula were inoculated in five BHI-HM mixtures and passaged once prior to metagenomic sequencing. Bar height represents normalized species relative abundance, and bars are colored by species. The x-axis represents the technical replicates (n = 3 technical replicates for 1 independent experiment). Source data are provided as a Source Data file.

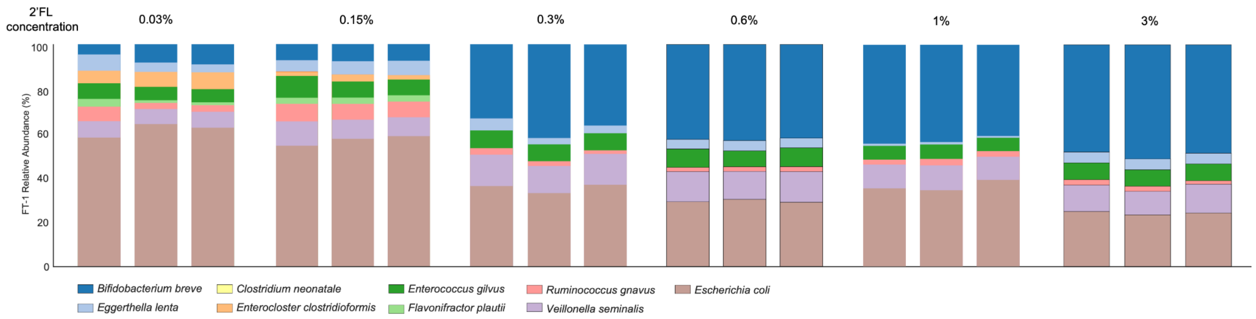

Supplementary Figure 5. The FT-1 microbiome grown in different concentrations of 2'FL
The compositions of the FT-1 enrichments grown in BHI-mucin supplemented with one passage with varying concentrations of 2'FL (0.03%-3%) are shown in stacked bar plots. Bar height represents normalized species relative abundance, and bars are colored by species. The x-axis represents the growth media, and all replicates are shown (n = 3 technical replicates for each experiment with a specific 2'FL concentration). Source data are provided as a Source Data file.

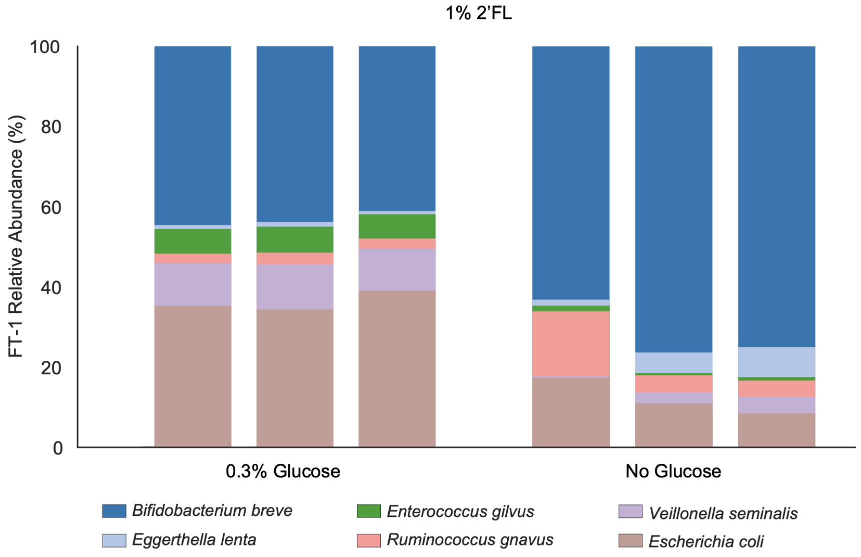

Supplementary Figure 6. Glucose concentrations influence the FT-1 microbiome composition

The compositions of the FT-1 enrichments grown in BHI-mucin supplemented with 1% 2’FL with (“0.3% Glucose”) and without (“No Glucose”) glucose. Communities were passaged once prior to harvesting for sequencing. Bar height represents normalized species relative abundance, and bars are colored by species. The x-axis represents the growth media, and all replicates are shown (n = 3 technical replicates for 1 independent experiment). Source data are provided as a Source Data file.

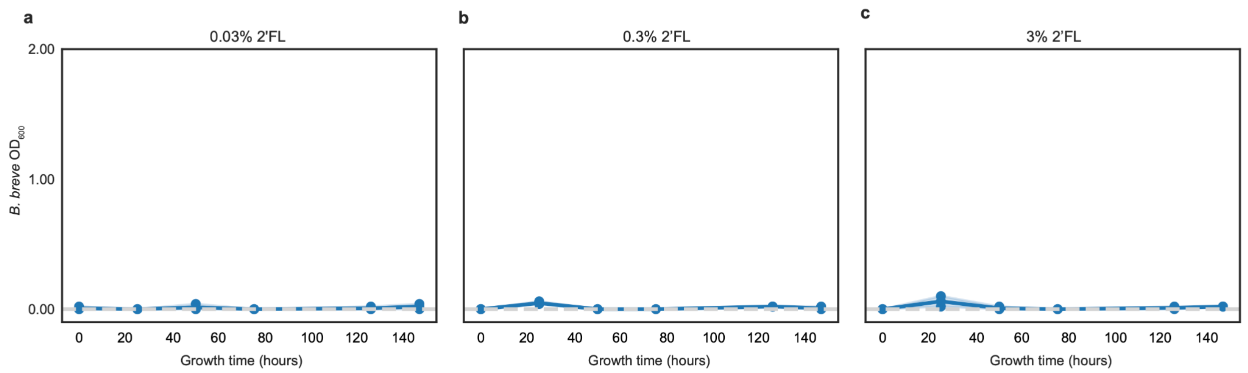

Supplementary Figure 7. *B. breve* monoculture growth in 2’FL

B. breve (blue line) grew in media in which 0.03% (**a**), 0.3% (**b**), or 3% (**c**) 2’FL was added as the only carbohydrate source. The x-axis represents the growth time (in hours). Each circle represents a replicate sample, and the line represents the average of all replicates (n = 3 for 1 independent experiment) at each time point; error bands indicate the 95% confidence interval. Source data are provided as a Source Data file.

Supplementary Table 1: FT-1 organism abundance comparison between enrichments that underwent one and three passages in media containing 0.4% or 0.6% mucin. Two-sided Welch's t-tests were run to compare the organism abundance in enrichments undergoing one and three passages. *P*-values were adjusted for multiple comparisons using Benjamini–Hochberg correction to get q-values.

Condition	Species	Stats	p-value	q-value
**BHI+0.4% mucin**				
BHI+0.4% mucin	*Bifidobacterium breve*	1.69E+01	2.40E-03	3.60E-02
BHI+0.4% mucin	*Eggerthella lenta*	3.64E-02	9.74E-01	9.74E-01
BHI+0.4% mucin	*Enterococcus gilvus*	-1.69E+00	1.71E-01	3.35E-01
BHI+0.4% mucin	*Escherichia coli*	3.51E-01	7.58E-01	8.12E-01
BHI+0.4% mucin	*Flavonifractor plautii*	-1.78E+00	2.16E-01	3.60E-01
BHI+0.4% mucin	*Ruminococcus gnavus*	-1.30E+00	2.99E-01	4.08E-01
BHI+0.4% mucin	*Veillonella seminalis*	-3.23E+00	8.03E-02	2.01E-01
**BHI+0.6% mucin**				
BHI+0.6% mucin	*Bifidobacterium breve*	3.42E+00	6.92E-02	2.01E-01
BHI+0.6% mucin	*Clostridium neonatale*	-9.91E-01	5.01E-01	6.26E-01
BHI+0.6% mucin	*Eggerthella lenta*	-1.63E+00	1.79E-01	3.35E-01
BHI+0.6% mucin	*Enterococcus gilvus*	-5.89E+00	2.25E-02	1.13E-01
BHI+0.6% mucin	*Escherichia coli*	3.66E+00	2.16E-02	1.13E-01
BHI+0.6% mucin	*Flavonifractor plautii*	-3.98E+00	5.61E-02	2.01E-01
BHI+0.6% mucin	*Ruminococcus gnavus*	-4.61E-01	6.88E-01	7.94E-01
BHI+0.6% mucin	*Veillonella seminalis*	-1.43E+00	2.83E-01	4.08E-01

Supplementary Table 2. Spearman correlation between inoculum organism abundance and 2'FL concentrations. Spearman correlation analyses were run to investigate the changes in organism abundance in relation to 2'FL concentrations (0%, 0.03%, and 0.3%). *P*-values were adjusted for multiple comparisons using Benjamini–Hochberg correction to get q-values.

Infant sample	Species	Correlation	p-value	q-value
FT-1	*Bifidobacterium breve*	8.50E-01	5.89E-05	1.38E-04
FT-1	*Escherichia coli*	-8.50E-01	5.89E-05	1.38E-04
FT-1	*Veillonella seminalis*	8.50E-01	5.89E-05	1.38E-04
FT-1	*Enterococcus gilvus*	-4.09E-01	1.30E-01	2.27E-01
FT-1	*Ruminococcus gnavus*	-3.78E-01	1.65E-01	2.31E-01
FT-1	*Eggerthella lenta*	-2.52E-01	3.65E-01	3.65E-01
FT-2	*Bifidobacterium breve*	8.45E-01	8.24E-03	3.57E-02
FT-2	*Enterococcus faecalis*	-8.45E-01	8.24E-03	3.57E-02
FT-2	*Veillonella parvula*	-8.45E-01	8.24E-03	3.57E-02
FT-2	*Clostridium* sp. 7_2_43FAA	-6.20E-01	1.01E-01	2.19E-01
FT-2	*Enterococcus avium*	6.20E-01	1.01E-01	2.19E-01
FT-2	*UNK Faecalimonas species*	-6.20E-01	1.01E-01	2.19E-01
FT-2	*Citrobacter braakii*	-5.67E-01	1.43E-01	2.57E-01
FT-2	*Enterobacter cloacae*	-6.55E-01	1.58E-01	2.57E-01
FT-2	*Enterococcus gallinarum*	2.82E-01	4.99E-01	7.21E-01
FT-2	*Anaerococcus vaginalis*	1.69E-01	6.89E-01	8.95E-01
FT-2	*Citrobacter freundii*	-1.31E-01	8.05E-01	8.95E-01
FT-2	*Clostridium perfringens*	5.63E-02	8.95E-01	8.95E-01
FT-2	*Klebsiella quasivariicola*	-5.63E-02	8.95E-01	8.95E-01
PT-1	*Proteus mirabilis*	-8.78E-01	2.13E-02	1.28E-01
PT-1	*Enterobacter hormaechei*	5.07E-01	2.00E-01	4.51E-01
PT-1	*Bifidobacterium breve*	7.75E-01	2.25E-01	4.51E-01
PT-1	*Clostridioides difficile*	-5.63E-02	8.95E-01	8.95E-01
PT-1	*Escherichia coli*	-5.63E-02	8.95E-01	8.95E-01
PT-1	*Klebsiella oxytoca*	5.63E-02	8.95E-01	8.95E-01

Supplementary Table 3. Mono- and co-culture growth metrics.

Condition	Culture	Growth rate (h^{-1})	Final OD_{600} mean	Final OD_{600} std
BHI (Glu-) + 1% 2'FL	*B. breve* monoculture	0.02	0.05	0.00
BHI (Glu-) + 1% 2'FL	*R. gnavus* monoculture	0.12	0.91	0.39
BHI (Glu-) + 1% 2'FL	Co-culture	0.18	1.22	0.12
BHI (Glu-) + 1% Fucose	*B. breve* monoculture	0.07	0.08	0.00
BHI (Glu-) + 1% Fucose	*R. gnavus* monoculture	0.13	0.76	0.06
BHI (Glu-) + 1% Fucose	Co-culture	0.14	0.73	0.04
BHI (Glu-) + 1% Lactose	*B. breve* monoculture	0.18	1.70	0.14
BHI (Glu-) + 1% Lactose	*R. gnavus* monoculture	0.11	1.18	0.74
BHI (Glu-) + 1% Lactose	Co-culture	0.18	1.65	0.07
