# Supplementary material for: Infant microbiome cultivation and metagenomic analysis reveal Bifidobacterium 2’-fucosyllactose utilization can be facilitated by coexisting species
Source: Nat Commun. 2023 Nov 16;14:7417. doi: 10.1038/s41467-023-43279-y (PMC10654741; doi:10.1038/s41467-023-43279-y)
Supplement: Supplementary file 3 — Description of Additional Supplementary Files [file 41467_2023_43279_MOESM3_ESM.pdf]

Description of Additional Supplementary Files

Infant microbiome cultivation and metagenomic analysis reveal *Bifidobacterium* 2’-fucosyllactose utilization can be facilitated by coexisting species

Lou et al.

Supplementary Data File 1. 2'FL-metabolism relevant genes and their presence/absence in FT-1 organisms.

Supplementary Data File 2. Key CAZymes and their presence/absence in organisms from different infant stool inocula.

Source data. All datasheets used for generating the main and the supplementary figures are included in the source data Excel sheet.
