# Supplementary material for: Infant microbiome cultivation and metagenomic analysis reveal Bifidobacterium 2’-fucosyllactose utilization can be facilitated by coexisting species
Source: Nat Commun. 2023 Nov 16;14:7417. doi: 10.1038/s41467-023-43279-y (PMC10654741; doi:10.1038/s41467-023-43279-y)
Supplement: Supplementary file 6 — Reporting Summary [file 41467_2023_43279_MOESM6_ESM.pdf]

nature portfolio

Corresponding author(s): Jillian F. Banfield

Last updated by author(s): Nov. 2, 2023

Reporting Summary

Nature Portfolio wishes to improve the reproducibility of the work that we publish. This form provides structure for consistency and transparency in reporting. For further information on Nature Portfolio policies, see our Editorial Policies and the Editorial Policy Checklist.

Statistics

For all statistical analyses, confirm that the following items are present in the figure legend, table legend, main text, or Methods section.

n/a	Confirmed	
☐	☒	The exact sample size (n) for each experimental group/condition, given as a discrete number and unit of measurement
☐	☒	A statement on whether measurements were taken from distinct samples or whether the same sample was measured repeatedly
☐	☒	The statistical test(s) used AND whether they are one- or two-sided<br>*Only common tests should be described solely by name; describe more complex techniques in the Methods section.*
☒	☐	A description of all covariates tested
☐	☒	A description of any assumptions or corrections, such as tests of normality and adjustment for multiple comparisons
☐	☒	A full description of the statistical parameters including central tendency (e.g. means) or other basic estimates (e.g. regression coefficient) AND variation (e.g. standard deviation) or associated estimates of uncertainty (e.g. confidence intervals)
☐	☒	For null hypothesis testing, the test statistic (e.g. F, t, r) with confidence intervals, effect sizes, degrees of freedom and P value noted<br>*Give P values as exact values whenever suitable.*
☒	☐	For Bayesian analysis, information on the choice of priors and Markov chain Monte Carlo settings
☒	☐	For hierarchical and complex designs, identification of the appropriate level for tests and full reporting of outcomes
☒	☐	Estimates of effect sizes (e.g. Cohen's d, Pearson's r), indicating how they were calculated

Our web collection on statistics for biologists contains articles on many of the points above.

Software and code

Policy information about availability of computer code

Data collection	bcl2fastq v2.20<br>Sickle (www.github.com/najoshi/sickle)<br>Bowtie2 v2.3.5.1<br>IDBA-UD v1.1.3<br>MetaBAT v2.12.1<br>CONCOCT v1.1.0<br>MaxBin v2.2.7<br>DasTool v1.1.1<br>dRep v3.2.2
Data analysis	Prodigal v2.6.3<br>USEARCH v10.0.240<br>tRep (https://github.com/MrOlm/tRep/tree/master/bin)<br>GTDB-Tk v2.2.6<br>inStrain v1.5.1<br>kofamscan v1.3<br>run_dbcan v4.0.0<br>HMMER v3.3.2<br>cath-resolve-hits v0.16.5

BLASTP v2.12.0+
SignalP v.5.0b
Python v3.9.1
Seaborn v0.11.1
Matplotlib v3.4.2
SciPy v1.6.3
scikit-bio v0.5.6
scikit-learn v1.3.2

For manuscripts utilizing custom algorithms or software that are central to the research but not yet described in published literature, software must be made available to editors and reviewers. We strongly encourage code deposition in a community repository (e.g. GitHub). See the Nature Portfolio guidelines for submitting code & software for further information.

Data

Policy information about availability of data

All manuscripts must include a data availability statement. This statement should provide the following information, where applicable:

- Accession codes, unique identifiers, or web links for publicly available datasets
- A description of any restrictions on data availability
- For clinical datasets or third party data, please ensure that the statement adheres to our policy

The metagenome-assembled genomes data generated in this study have been deposited on Figshare under accession code 10.6084/m9.figshare.22320865 (https://figshare.com/articles/dataset/Lou_et_al_2023_bioRxiv_de_novo_bacterial_genomes/22320865). Reads of the infant stool inocula are available under SRA accessions SRS8184257 (FT-1; L2_031_090G1) (https://www.ncbi.nlm.nih.gov/biosample/?term=SRS8184257%20), SRS8184183 (FT-2; L3_130_056G1) (https://www.ncbi.nlm.nih.gov/biosample/?term=SRS8184183), and SRS8184427 (PT-1; L3_069_015G1) (https://www.ncbi.nlm.nih.gov/biosample/?term=SRS8184427). All data used for generating the main and supplementary figures are provided in the Source Data. The 1005 representative subspecies used for this study's read-mapping-based species detection were generated from our previous study45 and are available on Figshare: 10.6084/m9.figshare.13667816 (https://figshare.com/articles/online_resource/dRepGenomes_Lou2021_tar_gz/13667816). The UniRef100, Pfam r35, NCBI BLASTP, and the Transporter Classification Database (TCDB) databases used for annotation in this study are publicly available (https://www.uniprot.org/help/uniref, http://pfam.xfam.org/, https://ftp.ncbi.nlm.nih.gov/blast/ and https://www.tcdb.org/, respectively).

Research involving human participants, their data, or biological material

Policy information about studies with human participants or human data. See also policy information about sex, gender (identity/presentation), and sexual orientation and race, ethnicity and racism.

Reporting on sex and gender	FT-1 and FT-2 were collected from males and PT-1 was collected from a female. This information was reported by parents during stool sample collection (see Lou et al.2021 (https://doi.org/10.1016/j.xcrm.2021.100393)). For this study, sex and gender were not considered. Sample selection was solely based on the microbiome composition. Refer to Lou et al.2021 for more detailed information on infant metadata.
Reporting on race, ethnicity, or other socially relevant groupings	FT-1 and FT-2 were from Caucasian infants and PT-1 was from an African American infant. This information was reported by parents during stool sample collection (see Lou et al.2021 (https://doi.org/10.1016/j.xcrm.2021.100393)). For this study, race, ethnicity, and other socially relevant groupings were not considered. Sample selection was solely based on the microbiome composition. Refer to Lou et al.2021 for more detailed information on infant metadata.
Population characteristics	All infants were breastfed only and biologically unrelated to one another. Their stool samples were collected before infants turned 3-month-old and their gut microbiomes shared one near-identical Bifidobacterium breve strain. Refer to Lou et al.2021 (https://doi.org/10.1016/j.xcrm.2021.100393) for more detailed information on infant metadata.
Recruitment	Refer to Lou et al.2021 (https://doi.org/10.1016/j.xcrm.2021.100393) for detailed information on infant enrollment and stool collection. This information is irrelevant to this current study. As stated above, stool sample selection was solely based on the microbiome composition, which was analyzed in Lou et al.2021.
Ethics oversight	This study was reviewed and approved by the University of Pittsburgh Human Research Protection Office (IRB STUDY19120040). All infant stool samples were collected with parental consent and subjects were de-identified before the receipt of samples. Refer to Lou et al.2021 (https://doi.org/10.1016/j.xcrm.2021.100393) for detailed information on infant enrollment and stool collection.

Note that full information on the approval of the study protocol must also be provided in the manuscript.

Field-specific reporting

Please select the one below that is the best fit for your research. If you are not sure, read the appropriate sections before making your selection.

[x] Life sciences   [ ] Behavioural & social sciences   [ ] Ecological, evolutionary & environmental sciences

For a reference copy of the document with all sections, see nature.com/documents/nr-reporting-summary-flat.pdf

Life sciences study design

All studies must disclose on these points even when the disclosure is negative.

Sample size	No calculations were done to determine the sample size. This study was designed to mechanistically investigate how compositionally and functionally distinct infant gut microbiomes would respond to the 2'-fucosyllactose supplementation, and the primary approach was to use laboratory gut consortia. All experiments were conducted in at least three replicates and the results among replicates were highly correlated, suggesting the reproducibility of our data. The three infant stool samples were selected based on the microbiome composition (all contain Bifidobacterium breve and no other Bifidobacterium strains) and, to a lesser extent, the stool availability. Although only a total of three infant gut consortia were examined in this paper, the key finding that the presence of extracellular-fucosidase-encoding community organisms can promote the extensive growth of B. breve was seen in all of them. We thus conclude that our sample size is sufficient.
Data exclusions	All experimental data points were included in this study. For organisms, if they were not detected in the initial inoculum and were present in <40% of the replicates across all tested growth conditions, they were defined as contaminants and were removed from the final data tables.
Replication	All growth conditions were performed in at least three technical replicates. For the chosen base condition (BHI+0.4% mucin for FT-1 and BHI +0.6% mucin for FT-2 and PT1), the experiments were repeated at least twice to confirm the reproducibility of the growth medium for the given infant stool inoculum. All technical replicates and independent experiments (replications) were reported.
Randomization	This is not relevant to our study. All work was conducted with controlled experiments in which it is expected that only the independent variable will differ between control and experimental groups.
Blinding	This study has some degree of blinding. During experimental processing (DNA extraction and library preparation), authors were not fully aware of the sample group allocation. In addition, the initial bioinformatics data processing was done without the authors being aware of the sample identities. The sample identity were reassigned in the final stages of data processing and analyses.

Reporting for specific materials, systems and methods

We require information from authors about some types of materials, experimental systems and methods used in many studies. Here, indicate whether each material, system or method listed is relevant to your study. If you are not sure if a list item applies to your research, read the appropriate section before selecting a response.

Materials & experimental systems

n/a	Involved in the study
☒	☐ Antibodies
☒	☐ Eukaryotic cell lines
☒	☐ Palaeontology and archaeology
☒	☐ Animals and other organisms
☒	☐ Clinical data
☒	☐ Dual use research of concern
☒	☐ Plants

Methods

n/a	Involved in the study
☒	☐ ChIP-seq
☒	☐ Flow cytometry
☒	☐ MRI-based neuroimaging

Plants

Seed stocks	*Report on the source of all seed stocks or other plant material used. If applicable, state the seed stock centre and catalogue number. If plant specimens were collected from the field, describe the collection location, date and sampling procedures.*
Novel plant genotypes	*Describe the methods by which all novel plant genotypes were produced. This includes those generated by transgenic approaches, gene editing, chemical/radiation-based mutagenesis and hybridization. For transgenic lines, describe the transformation method, the number of independent lines analyzed and the generation upon which experiments were performed. For gene-edited lines, describe the editor used, the endogenous sequence targeted for editing, the targeting guide RNA sequence (if applicable) and how the editor was applied.*
Authentication	*Describe any authentication procedures for each seed stock used or novel genotype generated. Describe any experiments used to assess the effect of a mutation and, where applicable, how potential secondary effects (e.g. second site T-DNA insertions, mosiacism, off-target gene editing) were examined.*
